# Impact of Web-Based Sharing and Viewing of Self-Harm–Related Videos and Photographs on Young People: Systematic Review

**DOI:** 10.2196/18048

**Published:** 2021-03-19

**Authors:** Amanda Marchant, Keith Hawton, Lauren Burns, Anne Stewart, Ann John

**Affiliations:** 1 Swansea University Medical School Swansea United Kingdom; 2 Centre for Suicide Research University Department of Psychiatry Oxford United Kingdom

**Keywords:** self-harm, suicide, social media, internet, systematic review

## Abstract

**Background:**

Given recent moves to remove or blur self-harm imagery or content on the web, it is important to understand the impact of posting, viewing, and reposting self-harm images on young people.

**Objective:**

The aim of this study is to systematically review research related to the emotional and behavioral impact on children and young people who view or share web-based self-harm–related videos or images.

**Methods:**

We searched databases (including Embase, PsychINFO, and MEDLINE) from January 1991 to February 2019. Search terms were categorized into internet use, images nonspecific and specific to the internet, and self-harm and suicide. Stepwise screening against specified criteria and data extraction were completed by two independent reviewers. Eligible articles were quality assessed, and a narrative synthesis was conducted.

**Results:**

A total of 19 independent studies (20 articles) were included. Of these, 4 studies focused on images, 10 (11 articles) on videos, and 5 on both. There were 4 quantitative, 9 qualitative, and 7 mixed methods articles. In total, 11 articles were rated as high quality. There has been an increase in graphic self-harm imagery over time. Potentially harmful content congregated on platforms with little moderation, anonymity, and easy search functions for images. A range of reactions and intentions were reported in relation to posting or viewing images of self-harm: from empathy, a sense of solidarity, and the use of images to give or receive help to potentially harmful ones suggesting new methods, normalization, and exacerbation of self-harm. Viewing images as an alternative to self-harm or a creative outlet were regarded in 2 studies as positive impacts. Reactions of anger, hostility, and ambivalence have been reported. There was some evidence of the role of imitation and reinforcement, driven partly by the number of comments and wound severity, but this was not supported by time series analyses.

**Conclusions:**

Although the results of this review support concern related to safety and exacerbation of self-harm through viewing images of self-harm, there may be potential for positive impacts in some of those exposed. Future research should evaluate the effectiveness and potential harms of current posting restrictions, incorporate user perspectives, and develop recovery-oriented content. Clinicians assessing distressed young people should ask about internet use, including access to self-harm images, as part of their assessment.

## Introduction

### Background

Young people who self-harm engage in more web-based activities than others of a similar age [1]. There is a large presence of self-harm–related materials [2], such as blogs [3,4], large social media groups [5], thousands of highly viewed videos [6], websites [7], and dedicated online communities [8]. The nature of internet use is constantly evolving. Platforms such as Instagram and Tumblr have increased in popularity for self-harm communities, partly because they are image based [9]. A total of 44.00% (528/1200) of surveyed adolescents reported Instagram to be an important part of their daily lives [10], and internet searches for suicide are increasingly returning graphic imagery [11].

Mental imagery plays a role in determining future behaviors [12]. It is thought to be more emotionally evocative than verbal thoughts, with stronger links to affect [13]. Individuals who experience suicidal ideation often report *flash-forward* detailed imagery, similar to flashback imagery in posttraumatic stress disorder, related to suicidal acts [14,15]. Most youths who self-injured reported mental imagery when the urge to self-injure was strong [16,17]. Emotion-laden mental imagery of self-harm may play a role in motivating behaviors toward or avoiding self-harm [17].

In addition to the role of mental imagery, a growing body of literature has explored the role of exposure to self-harm images through the media [8,18,19]. Although self-harm is a largely hidden behavior, images of self-harm are commonly shared on the internet. This is often done anonymously, with individuals continuing to hide their self-harm in the offline world [20]. Participants have reported reduced loneliness and the use of images to curb self-harm urges on the one hand and reinforcement and triggering of self-harm on the other [8]. Previous systematic reviews regarding internet use and self-harm, although not focused on images, similarly suggest both positive and negative impacts of images and videos [18,19]. The growing popularity of image-based platforms and high-profile stories of the risks has stimulated an exponential increase in research related to web-based self-harm imagery. Given recent moves to remove or blur self-harm imagery or content posted on platforms such as Instagram in response to concerns of bereaved parents [21], it is important to understand the impact of posting or viewing self-harm images on young people.

### Aims

This study aims to systematically review research related to the viewing or sharing of web-based self-harm or suicidal behavior–related videos or images in children and young people to explore the impact on emotions and behaviors of viewing or reposting images or videos of self-harm, the impact of posting images or videos of self-harm by individuals who self-harm, and whether certain aspects or types of images or videos impact outcomes.

## Methods

### Previous Reviews

Web-based content related to video and images of self-harm has been included as part of previous systematic reviews by our research group [18,19], but it was not our main focus. We adapted the search strategy used to specifically identify research on this topic. An electronic literature search was conducted from January 01, 1991, (the year the internet was made publicly available) to February 20, 2019, to ensure a comprehensive overview of the existing literature.

### Search Strategy

The core databases CINAHL, Embase (excluding MEDLINE journals), PsychINFO, SCOPUS, and MEDLINE were searched alongside topic-specific websites (Campbell, Centre for Mental Health, Department of Health, Mental Health Foundation, Department of Health, Social Services and Public Safety for Northern Ireland, National Health Service (NHS) Scotland, and the Royal College of Psychiatrists) and meta-search engines (Google and Google Scholar). The search terms were grouped into 4 categories:

1. Internet use: Free text “Aol” or “Askfm” or “Bebo” or “blog*” or “chat?room*” or “cyber*” or “discussion forum” or “e?communi*” or “e?material$” or “Facebook” or “googl*” or “hashtag$” or “instant messag*” or “internet” or “live chat*” or “live journal$” or “MSN” or “Myspace” or “on?line” or “online” or “podcast*” or “social network*” or “spam*” or “tweet*” or “Twitter” or “troll” or “virtual*” or “web” or “whatsapp”. *MeSH terms* “Internet,” “Social media,” “Social networking”
2. Images nonspecific to internet: Free text. “imag*” or “galler*” or “photo*” or “picture$” or “video*”. *MeSH terms* “Video recording”
3. Images specific to the internet: Free text. “meme” or “Pinterest” or “Tumblr” or “vine” or “vlog*” or “YouTube” or “snap*” or “gif$” or “selfie$” or “Flikr” or “camera” or “filter” or “reddit” or “Instagram” or “tik tok”
4. Self-harm and suicide: Free text. “Automutilation” or “Distress*” or “emotion*” or “NSSI” or “SIB” or “suicid*” or ((oneself or myself or self) adj2(cut* or harm* or hurt* or kill or injur* or mutilat*)). *MeSH terms* ‘Self-injurious behaviour’, ‘Stress, Psychological’

The terms were combined as follows (internet AND images nonspecific to internet terms) OR (images specific to the internet). The self-harm and suicide groups were combined with the results from the above search.

### Selection Criteria

Articles were included if they examined web-based viewing or sharing of images or videos related to self-harm or suicidal behavior or, videos and images viewed or shared by individuals who experienced suicidal ideation, suicidal behavior, or self-harm. Self-harm refers to any intentional act of self-injury or poisoning, independent of motivation or suicidal intent [22]. This definition is very general, as the motivation or degree of suicidal intent is difficult to assess and may vary between individuals and over time. Articles were required to include primary empirical data and be published in peer-reviewed journals. The results were not restricted by the location. Only English language articles were included. Review articles, single case studies, editorials, conference abstracts, or other grey literature were not included. The reference lists of all review articles were manually screened for potential eligible articles. Participants had to be aged 24 years or under or the study population had to have an average age of 24 years and under. If age was not specified, participants had to be described as children, adolescents, young people, or young adults. Where articles examined more than 1 age group, only data for the age group fitting these criteria were analyzed.

Two independent reviewers (AM and LB) manually screened titles. Any disagreements were resolved by consensus or discussed with a third expert reviewer (AJ). Duplicates were removed. Titles that had no relevance and gray literature were excluded from the title screen. A record was kept of all discarded articles, including the reason for exclusion. The remaining titles with abstracts were screened for eligibility (AM and LB). Full-text articles were obtained where suitability could not be determined based on the title and abstract.

### Data Analysis

A previously developed data extraction sheet [19] was used to record the findings (Multimedia Appendix 1). Additional fields were added to examine features specific to images and videos (eg, platform moderation, trigger warnings, the nature of the images or videos, and the impact on the viewer). Two reviewers (AM and LB) independently extracted the data from each article. Any inconsistencies in data extraction and quality scores were clarified by consensus or through discussion with a third expert reviewer (AJ).

The heterogeneous and largely qualitative nature of the studies precluded any meaningful combination of results through meta-analysis. Therefore, narrative synthesis was employed. On the basis of published guidance [23], this narrative synthesis examined a number of key aspects of each article. Comparisons were made regarding the way in which self-harm imagery was examined across studies and internet platforms. The influence of heterogeneity was explored further, including differences in populations studied, differences between various platforms, measures employed, and outcomes studied.

Articles were amalgamated and grouped into categories. These categories were inductively generated following initial reading and data extraction of articles and were cross-checked by members of the study team (AM, LB, and AJ). Positive influences of viewing or sharing of images and videos were defined as a perceived reduction in psychological distress, reduced suicidal ideation or self-harm, and advice on how to seek help and encouragement to do so. Negative influences were defined as results indicating increased psychological distress, increased frequency or severity of self-harm or suicidal ideation, provision of information on methods of self-harm or suicide, and self-harm behaviors being encouraged [19]. Positive and negative influences were examined in relation to the population studied, internet platforms (eg, Instagram and Tumblr), and specific features of images and videos.

The quality of the included articles was assessed according to the Critical Appraisal Skills Programme (CASP) [24], as described previously [19]. The CASP tool assesses various aspects of study design, including sources of bias, data collection, clarity of results, and appropriateness of conclusions. CASP does not specifically recommend any scoring or grading system. We adopted a scoring system used previously [25] (≤50% of criteria low quality, 50%-74% moderate, ≥75% high quality, and no criteria weighting applied [26]). Quality assessment was conducted by two independent reviewers (AM and LB) for each article.

A protocol for the systematic review was registered with PROSPERO (international prospective register of systematic reviews) [27].

## Results

### Screening Process

**Figure 1:**
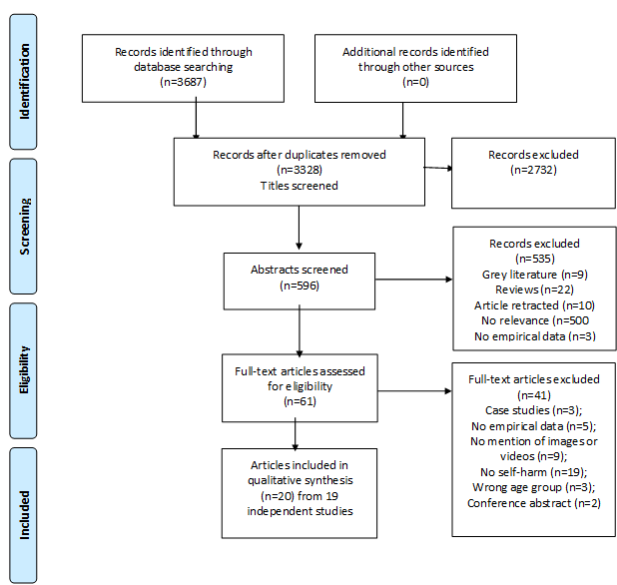
Results of the search strategy and screening process.

Figure 1 shows the results of the search strategy and screening process. A total of 19 independent studies (20 articles) were included. Of which, 4 studies focused on images, 10 studies (11 articles) on videos, and 5 on both images and videos. Two articles were based on a single data set of 100 videos [6,28]. A summary of the included articles, quality scores, by category of study is presented in Tables 1-3. Studies came from across the world (United States, n=7; Canada, n=4; United Kingdom, n=2; Germany, n=2; Australia, n=1; and multiple countries, n=3). There were 4 quantitative, 9 qualitative, and 7 mixed methods articles. In total, 11 articles were rated as high quality, 7 as moderate, and 2 as low. Tables 1-3 provide summaries of the included studies.

**Table 1:** Summary of included studies analyzing images or videos related to self-harm or suicidal behaviors.

Category (Author, year, country, quality score)	Analysis of images or videos related to self-harm or suicidal behaviors		
	Aims and objectives	Participants or sample	Results
Avery, 2015 [29], United States, moderate.	To understand the characteristics of the fire challenge phenomenon in an effort to develop preventative and public safety measures	50 videos depicting the fire challenge; 90% (45/50) male; 64% (32/50) African American	50 videos were selected. Of these, 13 videos included postburn footage. Superficial and partial thickness burns were sustained on the torso (10/13, 77%), face (4/13, 31%), and extremities (2/13, 15%). Full thickness burns were seen in 15% (2/13) videos. Young African American males were over-represented. The authors suggested that these should be targeted in prevention and intervention.
Basch, 2017 [30], United States, moderate.	To describe the extent to which content related to bullying is present on YouTube with respect to source, content, number of views, length, and year uploaded	The top 100 videos related to bullying on YouTube with the greatest number of total views were identified.	The most common content was describing or depicting violence (n=89). Over half addressed getting help (n=56). A total of 38 out of 100 videos mention suicide or thoughts of suicide. No analysis was conducted of the nature of discussions around suicide or their potential impact.
Brown, 2018 [31], Germany, high.	To analyze pictures directly depicting self-harm wounds on Instagram. Pictures, comments, and user accounts were examined. The aims were to systematically describe the extent of self-harm on Instagram in a German-speaking population and to describe web-based content of German-speaking users	Posters of self-harm pictures on Instagram (n=1152); majority anonymous; 91.0% (745/819) of identifiable profiles, female	Most pictures depicted wounds caused by cutting on arms or legs and were rated as mild or moderate injuries. Pictures with increasing wound grades and those depicting multiple methods of self-harm generated elevated amounts of comments. Most comments were neutral or empathic, with some offering help. Few comments were hostile. Pictures were mainly posted in the evening with a small peak in the early morning and on Sundays.
Cavazos-Rehg, 2016 [32], United States, high.	To gain a better understanding of the depression, self-harm, and suicidal content that is being shared on Tumblr	Tumblr accounts; of the 8 that provided demographic information 75% (6/8) were female.	A total of 17 Tumblr accounts posted a median number of 185 posts. Content was engaged with 1,677,362 times. Of the 3360 randomly selected posts, 81.52% (2739/3360) were related to depression, suicide, or self-harm. Common themes were self-loathing (412/2739, 15.04%), loneliness or feeling unloved (405/2739, 14.79%), self-harm (407/2739, 14.86%), and suicide (372/2739, 13.58%). Findings signal a need for suicide prevention efforts to intervene on Tumblr and use this platform in a strategic way.
Duggan, 2012 [2], Canada, low.	To simultaneously examine the scope and nature of self-harm content across informational or interactive websites, social networking websites, and YouTube to provide mental health practitioners with a multifaceted description of web-based content related to self-harm	Demographics only available for uploaders of YouTube videos; 80% (4/5) were female.	Peer-driven, informal websites have a variety of triggering content and are accessed more often than professionally driven sites. Self-harm is strongly represented among social networking websites and YouTube, evidenced by large group memberships and large video view counts.
Grzanka, 2014 [33], United States, low.	To investigate the mass media “It Gets Better” campaign responding to a perceived increase in suicides among gay youth in the United States since September 2010; critical discourse analysis of a sample of videos posted as part of campaign	Content of 30 videos aimed at LGBT+^a^ youth was analyzed.	Activists, celebrities, and the general public created and uploaded videos as part of the campaign, often telling their own stories of overcoming difficulties (eg, bullying). A thematic analysis of a subset of these videos revealed common themes of directives (eg, “don’t give up”) and testimonies of how the posters had overcome their own difficulties. The effectiveness of the campaign was not analyzed. Results revealed a neoliberal framing that placed the burden of a better life onto the emotional lives of LGBT+ youth, who were instructed to endure their pain in the interest of inevitable happiness.
Hilton, 2017 [34], United Kingdom, high.	To report the findings from a unique analysis of naturally occurring data regarding self-harm behavior generated through the global social media site, Twitter	Twitter users n=317; no information regarding gender	A total of 5 themes were identified: (1) celebrity influence, (2) self-harm is not a joke, (3) support for and from others, (4) eating disorders and self-harm, and (5) videos and personal stories. More recovery-oriented content than graphic imagery was found on this platform. No formal comparison with other platforms is made.
Lewis, 2012^b^ [28], Canada, high.	To examine viewers’ comments and responses to self-harm YouTube videos to determine the potential risks and benefits of the videos	Uploaders of 100 self-harm videos on YouTube; 95.0% (95/100) female	Most frequent comments were self-disclosure (334/869, 38.4%) and individuals sharing their own self-harm experiences. This was followed by feedback for the video uploader, including admiration of video quality (191/869, 22.0%) or message (148/869, 17.0%), and admiration for uploader (134/869, 15.4%), or encouragement for uploader (97/869, 11.2%). Many of those self-disclosing did not mention recovery (374/869, 43.0%) and indicated they were still self-injuring (295/869, 34.0%).
Lewis, 2011^b^ [6], Canada, moderate.	To explore the accessibility and scope of web-based self-harm videos	Uploaders of 100 self-harm videos on YouTube; 95.0% (95/100) female	The top 100 videos analyzed were viewed over 2 million times, and 80.0% (80/100) were accessible to a general audience. Viewers rated the videos positively and selected videos as a favorite over 12,000 times. The tone of the videos was largely factual or educational (53/100, 53.0%) or melancholic (eg, hopeless statements, depictions of sadness or crying; 51.0% [51/100]). Explicit imagery of self-injury was common. A total of 90.0% (90/100) of noncharacter videos had self-harm photos, whereas 28.0% (28/100) of character videos had in-action self-harm. For both, cutting was the most common method. Many videos (58/100, 58.0%) did not warn about this content. Content was often creative and frequently contained graphic imagery.
Lewis, 2015 [35], Canada, high.	To examine the nature of self-harm first aid tip videos shared through YouTube	40 uploaders of videos to YouTube; 83% (33/40) female	A total of 40 self-harm YouTube videos were content-analyzed. Videos were viewed 157,571 times and typically favorably rated. Most had a neutral purpose and neither encouraged nor discouraged self-harm. Messages encouraging self-harm help seeking were scant. Medical help seeking was not commonly encouraged 55% (22/40) of videos related to self-cutting and 28% (11/40), related to self-burning recommended seeking medical help, with several videos providing “safe” self-harm instructions.
Moreno, 2016 [36], United States, high.	To evaluate the meaning, popularity, and content advisory warnings relating to ambiguous self-harm hashtags on Instagram by (1) presenting current data on ambiguous hashtags that may be commonly related to self-harm; (2) testing a process to investigate ambiguous self-harm terms; (3) evaluating the popularity of self-harm-related hashtags at 2 time points; and (4) assessing the precision of Instagram’s warning labels for concerning content	Posters of ambiguous self-harm hashtags on Instagram; 193 unique usernames in a sample of 201 posts; no information on gender	Sample of 201 Instagram posts led to identification of 10 ambiguous self-harm hashtags. Results demonstrated a popular image that described the broader community of self-harm and mental illness called #mysecretfamily that had approximately 900,000 search results at time 1 and 1.5 million at time 2. Only one-third of relevant hashtags generated content advisory warnings.
Poonai, 2018 [37], Canada, high.	To use interrupted time series analysis to examine whether the release of Amanda Todd’s YouTube video following her death announcement in October 2012 was associated with an increase in average monthly emergency department visit rates for suicide-related diagnoses in Ontario children aged 11-17 years	Population-based time series analysis using a national database. Ontario patients aged 11-17 years based on a sample of 4,775,658 emergency department visits; 48.87% (2,333,822/4,775,658) female	There was a significant increase in the monthly emergency department visit rate for the composite outcome of the average monthly rate of initial emergency department visits for suicidal ideation, intentional self-poisoning, and intentional self-harm. Secondary outcomes were average monthly rates of death or intensive care unit admission resulting from index visit from April 2002 to December 2013. There was no significant change in emergency department visit rate for the composite outcome before and after announcement of Amanda Todd’s death. Findings suggest publicity around this video release was not associated with increase in emergency department visits for suicidal behavior.
Singaravelu, 2015 [7], United Kingdom, high.	To identify and analyze websites potentially accessed by young people about self-harm	Searched only for websites where target audience were described as young people. Initial search terms were developed in discussion with 6 members of a Child and Adolescent Mental Health Services users group aged 15-19 years.	Sites accessed by self-harm or suicide search terms were mostly positive or preventative in tone, whereas sites accessed by the term “ways to kill yourself” tended to have a negative tone. A total of 314 websites were included in analysis, with evocative images of self-harm found in 21.0% (66/314) of sites.

^a^LGBT+: Lesbian, gay, bisexual, transgender, and others.

^b^Analysis of the same dataset of YouTube videos with one study examining the content of videos themselves and the other analyzing the comments.

**Table 2:** Summary of included studies examining the perspectives of individuals on the impact of sharing and viewing web-based images or videos of self-harm.

Category (Author, year, country, quality score)	User perspectives		
	Aims and objectives	Participants or sample	Results
Jacob, 2017 [9], United Kingdom, high.	To explore how young people understand and use web-based images of self-harm using semistructured interviews	21 individuals aged 16-24 years, living in Wales, United Kingdom, with a previous history of self-harm. Mean age for self-harm commencement was 13 years. A total of 16 participants sought professional help, 8 presented to emergency departments for their injuries; 86% (18/21) were female.	Some individuals cited the internet as a catalyst in the development of self-harm, where individuals were searching for advice and support and self-harm had “just come up” in the search with instructions and images. The majority engaged with web-based spaces to support and further develop a pre-existing set of self-harming practices and reported the role of the internet in normalizing self-harm. Image rather than text-based interactions were the primary reason cited for using the internet for self-harm–related purposes. Images were said to invoke a physical reaction and inspire behavioral enactment. Participants reported viewing self-harm images as part of a ritualistic practice.
Seko, 2015 [38], multiple countries, high.	To conduct qualitative analysis of web-based interviews with individuals who produce self-harm content. To understand why content creators create and share self-harm–themed content and what needs are met by doing so	Creators of self-harm creative content, n=17; 82% (14/17) female	Thematic analysis of participants’ narratives identified 2 prominent motives: self-oriented motivation (to express self and creativity, to reflect on self-harm experience, and to mitigate self-destructive urges) and social motivation (to support similar others, to seek out peers, and to raise social awareness). Participants also reported a double-edged impact of self-harm content both as a trigger and a deterrent to self-harm.
Sternudd, 2012 [39], multiple countries, moderate.	To examine discourses about self-injury photos from a user’s perspective using web-based questionnaires	Users of self-injury forum: n=52; 87% (45/52) female	Informants reported that viewing or sharing images had alleviating rather than triggering effects with production of images often about memory and proof. Publishing them was seen as a way of sharing experiences with others and to give or receive help. Self-injury photos were described as a resource of a self-harm community culture. Informants often emphasized that the outcome of viewing these photos varies due to individual and situational differences.

**Table 3:** Summary of included studies examining web-based interventions using videos.

Category (Author, year, country, quality score)	Web-based video interventions		
	Aims and objectives	Participants or sample	Results
Choi, 2016 [40], United States, moderate.	To determine the feasibility of using a specific video in a web-based suicide awareness program for Asian American and non-Hispanic White college students	University students: n=431; 78.0% (336/431) female	Asian Americans rated the suicide awareness video significantly lower for cultural relevance than non-Hispanic Whites. Collectivist cultural orientation was a significant predictor for cultural relevance, credibility, and appeal. Cultural orientation and race or ethnicity should be strongly considered when web-based suicide awareness programs are developed for college students.
Park, 2014 [41], United States, high.	To determine the predictors of watching a web-based suicide prevention video and whether data characteristics differed for those watching most or only part of the video	University students: n=650; 71.8% (467/650) female	When examining characteristics of individuals who watched a suicide prevention video (which included self-harm content), the video completion group included more females, undergraduates, and Asian Americans and had higher individualistic orientation and more correct manipulation check answers. The video noncompletion group skipped items in a purposeful manner, showed less interest in the video, and spent less time completing questionnaires.
Robinson, 2015 [42], Australia, moderate.	To examine the safety and acceptability of a web-based suicide prevention program and determine which components were found to be most helpful and enjoyable	Secondary school students: n=21; 81% (17/21) female	A total of 21 young people completed the intervention. Overall, the intervention did not lead to increases in suicidal ideation or distress. Participants reported enjoying the program, in particular watching the video diaries and completing the activities. Most participants said they would recommend the program to a friend.
Scherr, 2017 [43], Germany, moderate.	To examine the impact of a suicide awareness video on adherence to newspaper reporting guidelines; video intended to be used for web-based format	Journalism students: n=78; 69% (54/78) female	Awareness material exposure helped to improve responsible reporting of suicide, with the awareness video showing a stronger effect than written material alone.

### Categories of Studies

In total, 12 studies (13 articles) reported an analysis of images or videos related to self-harm or suicidal behaviors over a range of platforms (multiple sources, n=2; YouTube, n=6; Instagram, n=2; Tumblr, n=1; and Twitter, n=1). Three studies examined the perspectives of individuals on the impact of sharing and viewing web-based images or videos of self-harm [9,38,39]. Furthermore, 4 studies (1 high and 3 moderate quality) examined web-based interventions using videos [40-43]. All 4 studies reported the potential for positive impacts. Some studies [31] made a distinction between photographs of nonsuicidal self-harm and suicide attempts, but the criteria for these distinctions were often unclear, so the term self-harm is used throughout.

### Use of Hashtags

Moreno et al [36] examined the nature of ambiguous self-harm–related hashtags (eg, #selfharmmm). At the time of this study, Instagram’s terms of use (since revised [21]) blocked users from searching for the hashtag #selfharm. As a result, a number of ambiguous hashtags emerged to bypass the restrictions. The hashtag #selfharmmm was used to identify a number of other ambiguous hashtags linked to self-harm-related content. Such hashtags included #blithe, #selfinjury, and #ehtilb. The number of search results for each hashtag grew substantially over 150 days. Search results revealed a wider group of hashtags #mysecretfamily present across platforms, referring to a range of mental health issues, of which #cat represented self-harm.

### Ethical Approvals

Ethical approval was stated as unnecessary in 5 of the 13 articles examining content across platforms because of the public nature of the internet sources. Of the 13 articles, 3 articles stated that they had received institutional review board ethical approval and only 6 included any discussion of ethical issues, such as protecting individual identities or sensitivity in disseminating results, although the content was likely to have been created by potentially vulnerable children and young people.

### Platforms

#### Image-Based Platforms

Two studies examined self-harm content on Instagram [31,36]. Both found considerable image-related self-harm content. One examined photographs of self-harm over a 4-week period, investigating the nature of the pictures, associated comments, and timing of posting behavior [31]. Most photographs depicted wounds with mild or moderate severity. Pictures were generally posted in the evenings. Most comments on self-harm–related posts were part of a more general discussion (3291/6651, 49.47%), with 11.58% (770/6651) asking the user to stop self-harming [31]. One study examined Tumblr posts [32]. Posts that were comforting, supportive, or related to prevention made up 8.00% (220/2750) of all posts. Posts interacting with other users made up 9.05% (249/2750), of which 47.0% (117/249) provided emotional support and 51.0% (127/249) sought or provided advice. Of those seeking or providing advice, 40.9% (52/127) provided positive or supportive advice, whereas 25.1% (32/127) provided potentially harmful advice, including advice on concealment.

One qualitative interview study found that Tumblr was often reported as the preferred platform because of the ease of searching for and sharing of images, perceived anonymity, and lack of moderation compared with other social media sites. This freedom to access and post severe images of self-harm was reported to lead to the normalization and exacerbation of self-harm for some participants [9].

#### Video-Based Platforms

Seven studies (8 articles) [2,6,28,30,32-35] analyzed YouTube videos, of which 3 (4 articles) focused on self-harm–related videos [2,6,28,35]. Although visual representations of self-harm were common, two-fifths of YouTube videos in one study exhibited a message encouraging help seeking [2]. Analysis of 100 videos found that 53.0% (53/100) were educational, often discouraging self-harm (26/100, 26.0%) [6]. Comments on this set of videos mostly shared personal experiences (38/100, 38.0%) [28]. There were no significant differences in the types of comments based on the number of views, ratings, or types of videos. An examination of videos concerning self-harm first aid tips found 28% (11/40) of videos included a disclaimer indicating that self-harm was *acceptable* providing certain safety precautions were followed [35]. Other YouTube videos varied in content, including *fire challenge* videos in which an individual doused themselves in a flammable liquid, set themselves alight, and attempted to extinguish the flames before serious burns are inflicted [29]. This was 1 of only 2 studies [29,37] included in this review in which the majority of participants were male (45/50, 90%).

An analysis of Twitter posts found images and videos related to self-harm, but these were not graphic in nature [34] and included images of celebrity tattoos covering self-harm scars with the message *stay strong*. These images were shared with positive messages of growth and recovery. Video links tended to be of individuals sharing their own stories.

### Graphic Imagery

Images rather than text-based interactions have been reported as the primary reason for using the internet for self-harm purposes in semistructured interviews [9]. The nature of images was important in terms of outcomes. It was commonly expressed that *bluntly gruesome* photos [39] (eg, flesh, open wounds, and blood) were more triggering than pictures of scars and healing wounds [38]. Results from internet searches varied based on the search terms used, with the term *ways to kill yourself* eliciting a high proportion of sites with evocative images (20/43, 47%) [7]. Platforms vary according to the degree of graphic imagery. Visual representations of self-harm were common on YouTube. The majority of videos showed severe and open wounds and acute scarification, and 1 video portrayed 3 clips of suicide attempts [2]. Analysis of 100 videos similarly showed that images of self-harm were common (64/100, 64.0% of videos), with 28% (14/50) of character videos depicting live acts of self-harm [6]. A large number of self-harm images are posted daily on Instagram. Of 2826 self-harm images examined in one study, 39.60% (1119/2826) were rated as mild in severity (ie, superficial scratches), 47.78% (1350/2826) as moderate (ie, deeper cut and blood flowing), and 12.60% (356/2826) as severe (ie, very deep cut or a large amount of deeper cuts and blood), with 93.06% (2630/2826) of images depicting cuts [31]. In another study, 17.7% (127/718) of Tumblr posts on self-harm or suicide were graphic images or video clips [32].

A range of emotional reactions and impacts on the viewing or sharing of images or videos are described in Table 4.

**Table 4:** Summary of emotional reactions and impacts of viewing and sharing videos and images.

Impact of image or video		Findings reported
Anger or hostility		Spoof advertisement on Twitter for stick-on self-harm scars evoked reactions of anger and frustration [34] Some hostile comments about an uploader of self-harm content (57/864, 6.6%) were found in comments of YouTube videos [28] A small percentage of comments of self-harm–related Instagram posts were coded as abuse (450/6651, 6.77%) [31]
Other emotions		The reaction from viewing self-harm photographs varied considerably between informants and may be dependent on the individuals’ state of mind when they are viewed. A wide range of feelings were covered in responses, including being sad, sick, and shocked. Reactions such as depression, grief, and concern for themselves were stated [39] Common reactions reported after viewing a suicide prevention video included sadness, surprise, shock, and feeling overwhelmed. Almost 40% of participants indicated that they were most affected by the real, personal stories of family and friends of those who had taken their own lives (particularly the impact of suicide on the lives of the people left behind) or individuals who had survived a suicide attempt [40]
Ambivalence		Dramatic responses are not always reported when viewing self-injury photos, with some reporting it can be done to pass time [39]
**Exacerbation of self-harm urges or behavior**		
	Triggering	Nearly 3 quarters of interview participants reported that imagery (notably photographs) was the primary reason for their utilization of the internet, due to a powerful physical reaction that triggered the desire to self-harm. Reliance on the image as a trigger had led to images assuming a vital role within their ritualistic practice with “sessions” often commencing with retrieval of a web-based image. The power of the image primarily centered on their ability to *“bring back memories”* of previous self-harm or the ease with which they allowed the individual to envisage how others experience the act. Participants also reported looking at images deliberately to trigger more severe self-harm [9] Participants often discussed the triggering role of images. Many stated that whether an image would trigger an act depended on mood at the time. It was commonly expressed that photographs of flesh, open wounds with blood are more triggering than pictures of scars and healing wounds [38] About one-third of participants describe the outcome of viewing photographs as triggering, with “bluntly gruesome” photographs described as the most triggering content [39]
	Competition	Participants spoke of being inspired to recreate certain sets of practices presented by particular images. Discussion was characterized by a sense of competition with individuals desiring to emulate the depicted self-harm while chiding themselves when they failed to engage with more sophisticated and severe techniques [9]
	Imitation	Pictures depicting wounds generated around twice as many comments from users than pictures not depicting wounds. There was also a signiﬁcant association between wound grade and number of comments [31]. Time-related analyses did not support any effects of contagion or reinforcement [31,37] Participants reported that a lack of moderation on Tumblr and the freedom to view and share the most stark and severe images had led to normalization and exacerbation of self-harm. One participant stated that their self-harm had escalated from little gashes to severe injuries and cutting through arteries [9]
**Reduction in self-harm urges**		
	Calming	Participants reported self-harm photographs as providing a sense of vicarious relief and of viewing photos to calm themselves when feeling triggered [38]. Reactions to self-harm images were described as comforting or calming in nearly half of statements [39]
	Use as a deterrent	Some participants stated that self-harm photographs of severe injuries acted as a deterrent of self-harm. Participants reported using this as a pre-emptive strategy to avoid more severe self-injury [38]. One participant described viewing of severe injuries as making them feel nauseated, serving as a strategy to avoid more severe self-harm [38]
	Emotional outlet	Content creation, particularly artistic or creative content, was described as an emotional outlet to disclose negative emotions, distress, and aspects of the self otherwise difficult to express. This was reported, at times, to reduce self-harm urges by acting as a distraction or alternative to self-harm [38] Some participants reported that looking at content helped them reflect on their experience, make sense of it, and potentially avoid further episodes. Creation of content was reported to reduce self-harm urges serving as a creative alternative [38]. Participants reported viewing images made them feel less alone helping to curb NSSI^a^ urges. Feelings of relief were reported with photographs reducing urges to self-harm.
**Connection with others**		
	Empathy	Empathetic comments made up 23.49% (1562/6651) of comments on Instagram posts [31]. Many participants spoke of feeling empathy with content creators when viewing images related to self-harm. Several participants also addressed the internet as their only source of support or connection where they could receive empathetic reactions and emotional support [38]. Empathy and sympathy were common reactions to a suicide prevention video [40]
	Solidarity and reduction in loneliness	Participants reported the viewing of photographs as comforting, as they made them feel less alone [38,39]. Participants describe feeling less alone in their battle as a motivation for sharing images [39]
	Giving and receiving help	The motivation to support like-minded people was often described as going hand in hand with a desire for help [38] Warnings asking user to stop behavior were present in 11.58% (770/6651) comments on Instagram posts, as were offers of help (462/6651, 6.95%) [31] A total of 51.0% (127/249) of interactive Tumblr posts involve seeking or providing advice, of which 40.9% (52/127) provided positive support or advice (eg, encouragement in stopping self-harm) and 25.1% (32/127) provided potentially harmful advice (eg, advising how to secretly engage in self-harm), with 13.4% (17/127) suggesting professional help or therapy [32] An equal number of comments on YouTube videos asked for help or offered help (23/1150, 2.00%), and a small number encouraged the uploader to seek help (21/1150, 1.82%) [28] Participants who watched a suicide prevention video expressed a higher awareness of the need to watch for signs of depression to be able to help friends and the need to take immediate action, take depression seriously, and talk openly about suicide [40] A total of 9.09% (249/2739) of Tumblr posts involved directly interacting with another user. Of these, 47.0% (117/249) provided emotional support or reassuring messages to one another [32]
**Feedback or discussion of creative content**		Creators of creative content expected constructive criticism from their viewers to improve their artistic skill, and positive feelings were reported when content received comments or was reblogged [38] High levels of feedback were given or received for video content. Feedback included admiration of video quality (191/869, 22.0%) and the video message (148/869, 17.0%) and validation and admiration for the individual who uploaded the video (134/869, 15.4%) [28]. Very few Instagram comments complimented the wound or image (33/6651, 0.50%) [31]

^a^Nonsuicidal self-injury.

### Negative Reactions and Impacts

Anger and hostility in response to images or videos were reported in 3 studies [28,31,34]. Other emotional reactions included sadness, shock, and concern [39,40]. Some have described ambivalence or simply viewing photographs to pass time [39]. Images having the potential to exacerbate self-harm through triggering, normalization, competition, or imitation have also been described [9,38,39].

#### Triggering

The triggering role of imagery was reported in 3 qualitative interview studies [9,38,39]. A third of the participants reported that photographs triggered self-harm urges [39]. Participants reported viewing images as part of a ritualistic practice to be triggered before harming themselves to self-harm more severely [9]. Both triggering and alleviating effects of images have been reported [39]. Whether the material was triggering was dependent on the viewer’s mood. If an individual was already determined to self-harm, then looking at images would encourage urge, whereas if they were in a positive mood, the images would have minimal impact [38].

#### Normalization and Competition

There were reports of web-based engagement exacerbating self-harm due to normalization and exposure [9]. A sense of competition was reported in all 3 qualitative interview studies [9,38,39], with individuals desiring to emulate the depicted harm while chiding themselves or receiving negative comments from others when they failed to engage with more sophisticated and severe techniques [9]. Participants reported negative feelings regarding failure to harm themselves as severely as the injuries shown in photographs [38]. There were also reports of needing to make wounds worse to be a valid *self-injurer* [39]. A strong correlation between male informants and negative statements was found in 1 study, with 80% (4/5) of the statements expressing competitive reactions [39].

#### Imitation

Brown et al [31] examined several possible markers of imitative behavior on Instagram, including comments on photographs and a time-related analysis of posts. Pictures directly depicting wounds generated twice as many comments as those not depicting wounds [31]. A significant association was also observed between wound grade and number of comments, which could indicate a socially reinforcing function of posts. However, the time-related analysis of images did not indicate any effects of imitation or reinforcement [31]. A separate time series analysis did not find an increase in average monthly emergency department visits for suicide-related diagnoses in Ontario children aged 11-17 years following posting and publicity of a young person’s YouTube video who took their own life [37]. This showed the young person holding up cards telling a story of bullying, mental health issues, and self-harm, with an image of self-harm injuries at the end.

### Positive Reactions and Impacts

The production of images was often about memory and proof, with photos forming part of a narrative likened to fading scars. Individuals reported taking photographs for artistic value or their own interest [39]. A reduction in self-harm urges was reported with content creation said to act as an alternative outlet for negative emotions, with the viewing of images acting as a placebo or deterrent [38]. Those who create drawings, poems, and videos, in particular, called the materials a form of art that portrays the feelings behind self-harm, with positive feelings reported when content was reblogged [38].

Reactions of empathy, sympathy, and where people both gave and received help were found across various platforms [31,38,40]. Self-harm photographs were described as a resource for the self-harm community and were said to provide a feeling of solidarity and reassurance of not being alone [38,39].

### Prevention and Intervention

#### Trigger Warnings

Three studies (4 articles) exploring the content of images or videos included an analysis of trigger warnings. These are placed on content by either the uploader, moderator, or platform and are intended as a warning to individuals who may find that the images increase the urge to self-harm. Two articles (based on a single data set of 100 videos) reported that 58.0% (58/100) of YouTube videos related to self-harm did not warn about content [6,28]. Just 2 of 40 self-harm first aid videos contained such a warning [35]. Two peer-moderated and 3 professionally driven sites were examined in an assessment of self-harm across different platforms. Both peer-moderated sites contained trigger warnings. The professionally driven websites did not contain any graphic material, so no trigger warnings were present. Trigger warnings were present on 4 of the 5 YouTube videos included in this study. Of the 5 Myspace groups examined, 3 contained trigger warnings and 2 prohibited self-harm images. Only 1 of the 4 Facebook groups contained a trigger warning, despite the presence of photos and videos in most groups [2].

Alongside user-generated trigger warnings are content advisory warnings on some platforms. Searches for self-harm-related content on Tumblr first provided a screen with suggestions for seeking help or finding more inspirational content, with the option to proceed to the next screen to view the search results [32]. Of the 18 self-harm–related hashtags, 6 generated a warning label on Instagram [36].

#### Recovery-Oriented Content

Recovery-oriented content was observed in varying degrees across platforms. Among Tumblr posts providing advice, only 13.4% (17/127) suggested professional help or therapy [32], and 2.4% (21/869) of comments on YouTube videos encouraged the uploader to seek help [28]. Some comments indicated that the individual was seeking treatment (83/869, 9.6%), had recovered (62/869, 7.1%), or expressed a desire to recover (33/869, 3.7%) [28]. Recovery-oriented content appeared to be most evident on Twitter, with inspirational quotes and stories of recovery [34].

#### Interventions

The interventions discussed here include a web-based video component to an intervention designed to reduce suicide or self-harm. The content of these videos was professionally generated and differed from the user-generated content discussed above and contained no graphic self-harm imagery.

The safety and acceptability of a web-based suicide prevention program incorporating video elements in secondary school students was examined in one study [42]. The video component of this intervention uses a *host* character to deliver verbal therapy and parts of this include *video diaries* that tell a story portrayed with young people actors. Overall, the intervention did not lead to increases in suicidal ideation or distress, and 71.4% (15/21) of participants rated the video diary component as more enjoyable and helpful than hearing from website moderators or receiving text messages [42]. The impact of a suicide awareness video as part of a web-based intervention to increase adherence to newspaper reporting guidelines in journalism students was examined in another study. Exposure to awareness raising material with or without the video helped to improve responsible reporting of suicide, with the awareness raising video showing the strongest effect [43].

Two studies examined different aspects of *The truth about suicide* suicide awareness video. This video focused on interviews with people who have lost a loved one to suicide and the testimonials of students who have experienced depression and suicidal ideation. Both studies found video streaming to be a feasible method for delivering a suicide awareness program to college students [40,41]. Predictors of watching the 29-min video to completion were female gender, undergraduate, Asian ethnicity, and higher individualism as opposed to collectivism [41], measured using the individualism-collectivism questionnaire [44]. Asian American students rated the video significantly lower on cultural relevance than non-White Hispanic students [40]. Following viewing of the video, participants expressed a higher awareness of the signs of depression, the need to take immediate action, and to talk openly about suicide [40]. Small increases in distress were found [41]. The importance of an effective debriefing session in a safe and confidential setting for sharing thoughts, feelings, and experiences and to prevent iatrogenic effects of web-based suicide prevention programs using emotionally charged videos was emphasized and successfully delivered on the web [40].

## Discussion

### Principal Findings

We conducted a systematic review of the literature related to the impact of sharing or viewing web-based self-harm images or videos, exploring intent and interpretation in young people. A total of 19 studies (20 articles) were included. No previous studies have focused on this area [18,19]. We have identified specific features of platforms that contribute to potentially harmful use and of images that may influence the impact on the viewer or sharer.

In recent years, there has been a move away from the once popular self-harm forums to other platforms in terms of use by both individuals and researchers [9,45]. There has also been an increase in graphic imagery identified by self-harm–related searches over time [11], and participants reported image-based rather than textual interactions as the primary reason for internet use [9]. Studies consistently report a large presence of graphic imagery of self-harm on image-based platforms such as Instagram and Tumblr. In contrast, there is less graphic imagery present on Twitter and a greater presence of recovery-oriented content [34]. The difference between platforms was further supported by an analysis of randomly generated posts found with the hashtag #cutting. Instagram posts were found to display the greatest proportion of graphic content, with the smallest proportions found on Twitter [46]. Recovery-oriented resources were congregated on the platforms with the least problematic content. Potentially harmful content appeared to congregate on the platforms that have relatively little moderation, where participants can remain anonymous and search for images easily [9]. The use of videos in suicide prevention interventions has also been examined [40-43]. The content of these videos differed from user-generated materials, such as those shared on social media platforms. Videos included as part of an intervention focused on issues around suicide or self-harm, including interviews with those impacted by suicide [40,41], video diaries delivered by actors [42], and suicide awareness material related to response reporting of suicide by the press [43].

A range of reactions and intentions have been reported in relation to posting or viewing images of self-harm from empathy, solidarity, and the use of images to give or receive help [31,38] to potentially harmful advice suggesting new methods and tips for hiding self-harm, as well as normalization and exacerbation of self-harm [9,39]. This is further supported by recent research describing strong social motivations for posting self-harm content on Instagram as well as the need to document self-harm or recovery [47]. Viewing images as an alternative to self-harm and as a creative outlet were regarded in 2 studies as positive impacts [38,39]. The use of images as a creative outlet for distress was found in a recent analysis across platforms [48]. Reactions of anger, hostility, and ambivalence in posting self-harm images have also been reported [28,31,34].

Self-harm images were reported to be part of a ritualistic practice by all 3 studies that examined user perspectives. Participants reported viewing graphic imagery on the web to evoke the right mood for self-harm, often resulting in more severe injuries [9]. Photographs of fading scars or creative content have been reported to have a positive impact [38,39]. Photographs of fresh cuts or severe injuries are most frequently reported to have a negative impact [9]. Participants reported that viewing more severe injuries leads to negative feelings of failure to achieve the same level of injury or more severe injuries over time [9,39].

There was some evidence of the role of imitation and reinforcement, driven partly by the number of comments generated by self-harm imagery and wound severity [9,31]. Although time series analyses were conducted in 2 studies [31,37], the limitations of these analyses indicate the need for further studies to examine the impact of self-harm imagery. Although the evidence for social contagion is lacking in the papers described here, recent studies relating to the release of the TV series *13 Reasons Why,* in which the suicide of a young female is graphically depicted, found this to be associated with a significant increase in adolescent suicide rates [49] concordant with the period in which social media discussions of the series were greatest [50]. This increase in deaths was found despite some individuals reporting positive impacts [51]. The graphic scene was removed following concerns expressed by the suicide prevention agencies. Recent research has suggested a need to think beyond a model of contagion of self-harm behavior and shifting the focus onto other mechanisms of harm and benefit [52]. It has been highlighted that social media often acts to mirror society and that a wider context needs to be taken. For example, gaps in service provision often led people to seek online peer support [52].

### Strengths and Limitations

This study provides a comprehensive overview of studies directly examining web-based self-harm imagery or videos in relation to children and young people. The potential for publication bias exists in any literature review. Steps were taken to minimize bias, including conducting an extensive literature search of multiple databases, including topic-specific websites and reviewing reference lists, titles and abstracts being screened by 2 researchers, and data extraction being conducted by 2 researchers for each article. Only English language publications were included in the analysis. Females outnumber males, where this information was available, in all but 2 studies, one of videos related to the fire challenge [29] and a time series analysis of emergency department attendance [37]. One study reported that males were more likely than females to report negative impacts of self-harm images [39]. However, the limited number of males included makes it difficult to draw firm conclusions. Further research into potential sex differences and involving larger samples of males is needed. 

Although a number of measures were included in eligible studies, including analyses of content, the impact of self-harm imagery has relied heavily on self-reporting of short-term impacts. This is particularly true for the perceived positive impacts. Future research should aim to understand the long-term effects of continual exposure to such images, as the nature of immediate impacts may change over time. This is highlighted by the recent literature related to *13 Reasons Why,* where an increase in suicide rates was found [50], despite some individuals reporting a positive impact of the show [51]. Web-based content has the potential to reach a large audience; as such, a range of emotional reactions is to be expected. It is important that the overall impacts on outcomes such as suicide rates and levels of self-harm are assessed alongside self-report measures to put any reported positive or negative impacts into a wider context. The use of validated outcome measures related to self-harm or levels of distress would strengthen the evidence base in this area.

Although 2 studies included a time series analyses [31,37], both had their limitations. It is not yet clear how and whether any long-term process of imitation would take place on a platform such as Instagram, where images can be searched and viewed long after they are posted [31]. Although the potential impact of a particular video was examined for emergency department attendances, this was confined to 1 region that was not where the death occurred and media dissemination was greatest. This would not have been a measure of self-harm that presented to settings other than emergency departments, such as primary care, or for self-harm in the community that would not be present in health care data. Further research is needed before any conclusions can be drawn based on such evidence. This could include the use of survey data or routinely collected health care data spanning multiple settings (eg, primary and secondary care). Accurately determining the characteristics of young people based on web-based profiles is not always possible. The average age of participants is likely to be younger than that reported, as young people often misrepresent their age to gain access to restricted content [6]. Finally, although self-harm presentations by young people to general hospitals most commonly involve self-poisoning [53], this method of self-harm rarely featured in the images appearing on the internet. Thus, the images that are found largely reflect self-harming behavior most frequently occurring in young people in the community [53], although accessing internet sources before self-harm is common in young people who attend hospital following self-poisoning [54].

### Implications

Clinicians working with young people who self-harm should routinely enquire about internet use [54], support them in recognizing and managing triggering content, and encourage healthier web-based behaviors. The powerful role of imagery in evoking emotional reactions and motivating both potentially harmful behaviors, such as self-harm [16], and adaptive behavior [55] could make this an important factor in recovery.

As young people frequently create their own content, guidance to increase safe and responsible depiction of suicide and self-harm image and video-based content are now needed to educate young people as well as media professionals. Recent analysis of videos related to the *Blue Whale Challenge* on YouTube has demonstrated that although these user-generated videos seek to raise awareness, most videos violate guidance around safe and effective messaging, further underscoring the need to educate social media users and content generators on safe content creation and the factors that may have either positive or negative impacts on vulnerable viewers [56]. Individuals have reported an awareness of the potential impacts of self-harm images and videos and as a consequence add trigger warnings to their own posts [9]. Initiatives could be developed to build on this and to equip young people with the skills to analyze and evaluate their own creative content and to highlight the potential for posts intended as catharsis and therapy for themselves to be potentially harmful to vulnerable individuals. Strategies to improve safety could build on the work of the #chatsafe project and the development of guidelines for safe peer-to-peer web-based communication related to suicide [57]. Such projects could be used to inform parents, teachers, and other caregivers as well as young people. This could improve the safety of image-based platforms.

A small number of studies examined the use of videos disseminated on the web as a means of intervention. Given the large self-harm community using image and video-based platforms, further research into potential interventions is needed to understand how to maximize effectiveness. Content creation can act as an alternative outlet for negative emotions, and the viewing of images acting as a placebo or deterrent could be explored as potential interventions. It has been suggested that educational programs should be developed to inform young people about the impact of the content of posts and how to respond to posts from those in distress [2,57]. A recent pilot study found that hopeful reactions to the self-harm content of YouTube can improve positive attitudes toward recovery [58]. This has the potential to prevent suicide contagion via social media. The use of videos in suicide prevention programs appeared to be promising not just for young people themselves but also for improving responsible reporting of suicide among journalism students [43]. The importance of the cultural relevance of prevention videos has been emphasized [41]. More recent coproduction of suicide prevention material with a popular YouTuber in Hong Kong suggests that this may be an effective avenue for future suicide prevention strategies [59].

Individuals reported an awareness of the potential impacts of self-harm images and videos and added trigger warnings to their own posts [9]. Automated platform content warnings may not have been sufficient [36]. Our results should be discussed in the context of recent changes in policy by platforms, including Facebook [60] and Instagram [21], regarding the posting of graphic self-harm imagery. Changes were made following the death of a young person in the United Kingdom in February 2019 and the subsequent campaign by the family. Instagram announced a ban on all images of self-harm, including pictures showing scars [21]. The particular features of platforms that make them more amenable to harmful use include a lack of moderation and ease of sharing, searching, and viewing of images [9]. Monitoring and regulation of posts may be a positive move toward making these spaces safer. The effectiveness of such policies has yet to be evaluated, and a recent analysis of YouTube content found that content did not comply with YouTube’s policy on self-harm. Most content did not require age registration, and the specific help-related search terms had to be used to access any suicide prevention–related content [61]. Young people often find ways around posting restrictions, such as by using ambiguous hashtags [36] or moving to more hidden parts of the internet. An examination of 1 of these ambiguous hashtags on Instagram has shown that 3 quarters of posts contained a wound, the majority of which did not contain an advisory warning [62]. The use of image recognition software to identify graphic self-harm imagery could have important implications for protecting vulnerable individuals from potentially triggering content [63].

The potential for posting restrictions to reduce positive impacts, such as support and a sense of community, must also be considered as should any potential negative impacts of the removal of content posted by vulnerable young people without consultation. The use of social media to bridge support in service provision, particularly with regard to both giving and receiving peer support, has been further highlighted in recent literature, pointing to the potential inadequacy of a strategy focused on placing pressure on internet service providers to remove all self-harm content. Instead, care must be taken with such restrictions to avoid further harm [52]. Banning images of scars may have the unintended consequence of increasing stigma or removing from individuals the opportunity to share their stories, including those related to recovery. The indiscriminate removal or blurring of images of people with injuries and scars will include those not related to self-harm and could negate the potentially positive impact of fading or healing self-harm scars. Although the results of this review support concern related to safety and exacerbation of self-harm, the potential for positive impacts should not be underestimated. Future research should seek to evaluate the effectiveness of current posting restrictions and to identify the best strategies to reduce risk and maximize positive impacts on participants by incorporating user perspectives. This supports previous calls to better understand how to leverage the unique opportunities afforded by such platforms to reach and engage vulnerable individuals [46,47], with the potential for awareness materials to reach a large audience of vulnerable individuals via social media [64].

We identified no studies examining the role of the Google Images search engine, which has not seen the same restrictions applied to searches for self-harm imagery as social media platforms. There is no social community or moderation of Google images. Google Images does not incorporate the same tools that exist on the main Google search engine, ensuring results related to helplines, and information appears at the top of search results for suicide and self-harm. Typing *self-harm* into Google images results in a collection of images from across other sites. This is a powerful tool and appears not to have been the focus of this kind of research or policy intervention. It remains an ongoing challenge for the literature to remain current with the changing popularity of various platforms, for example, with the current popularity of Tik Tok, which does not appear to be the focus of current research.

It is important to note that the majority of studies reported here explore immediate or short-term reactions to self-harm content. Future research should aim to understand the long-term effects of continual exposure to such images, as the positive or negative immediate impacts may change over time.

Careful consideration should also be paid to the ethical issues of conducting research on freely available content and the associated implied consent. Future research should ensure the recruitment of males and focus on delineating the specific features of images that may be harmful and the mechanisms involved.

### Conclusions

The way in which young people use the internet is continually evolving. An increasing preference is being reported for the use of the internet for image-based rather than text-based interaction with regard to self-harm. Concerns over negative impacts such as exacerbation and normalization of self-harm and sharing of information on new methods or concealment of self-harm are supported by research. However, there can also be positive impacts such as seeking and receiving peer support and viewing images as an alternative to self-harm and as an outlet for negative feelings. Graphic images and photographs of severe injuries are most often reported to have a negative impact. Clinicians assessing distressed young people should routinely enquire about internet use, explore strategies to manage any triggering content, and be aware of helpful sites. The use of images and videos as part of web-based interventions for suicide prevention is an area worthy of further study. Future research should also seek to evaluate the effectiveness of current posting restrictions on social media and to educate clinicians and caregivers on how to encourage healthier web-based behaviors. The combination of appropriate policies by internet service providers and education for individuals may be necessary to allow for the potential positive impacts of web-based imagery while minimizing the potential for harm.

## Appendix

Multimedia Appendix 1 Data extraction form.

## Abbreviations

ADP: Adolescent Mental Health Data Platform
CASP: critical appraisal skills programme
NHS: National Health Service
