# Supplementary material for: Impact of Web-Based Sharing and Viewing of Self-Harm–Related Videos and Photographs on Young People: Systematic Review
Source: J Med Internet Res. 2021 Mar 19;23(3):e18048. doi: 10.2196/18048 (PMC8074996; doi:10.2196/18048)
Supplement: Multimedia Appendix 1 [file jmir_v23i3e18048_app1.docx]

**Multimedia Appendix 1.** Data extraction form.

|  | **Article Information** | **Pg. / Para / Table** |
| --- | --- | --- |
| Paper ID |  |  |
| Author |  |  |
| Year |  |  |
| Title |  |  |
| Type of study |  |  |
| Aims |  |  |
| Results |  |  |
| Participants Description |  |  |
| Participant Inclusion/Exclusion Criteria |  |  |
| Number of Participants |  |  |
| Age Range |  |  |
| Mean Age |  |  |
| Standard Deviation of Age |  |  |
| Percent Female |  |  |
| Were participants grouped? If so, how? |  |  |
| Locality |  |  |
| Country |  |  |
| Recruitment Method |  |  |
| Data Collection Method |  |  |
| Was the study quantitative, qualitative or mixed methods? |  |  |
| Time period of study |  |  |
| Specific Measures |  |  |
| Ethical Permission |  |  |
| Did the participants have a mental health diagnosis? |  |  |
| Diagnosis type/mental health subgroup |  |  |
| History of self-harm |  |  |
| Were participants divided into subgroups? E.g. self-harm, suicidal ideation |  |  |
| Were participants taking medication? |  |  |
| Family history of self-harm |  |  |
| Frequency use |  |  |
| Internet medium (e.g. social media, support sites etc.) |  |  |
| Platform (e.g. twitter, tumbler etc.) |  |  |
| Images, videos or both |  |  |
| Brief description of the images/videos |  |  |
| Number of images/videos |  |  |
| Images/videos inclusion criteria |  |  |
| Were any trigger warnings reported? |  |  |
| Were any help messages reported? |  |  |
| Any additional analysis of comments? |  |  |
| Description of findings related to comments |  |  |
| Were there any positive impacts? |  |  |
| Description of any positive findings |  |  |
| Were there any negative impacts? |  |  |
| Description of any negative findings |  |  |
| Findings that do not fall into positive or negative |  |  |
| Description of other findings |  |  |
| Was there any change in participants? |  |  |
| Suggested mechanism of change |  |  |
| Did participants give any reasons for use, sharing or viewing |  |  |
| Was the platform moderated? If so, was moderation peer or professional? |  |  |
| Describe any missing information |  |  |
| Notes |  | |
